# Human epidermal growth receptor polymorphisms (*HER1*–rs11543848 and *HER2*–rs1136201) exhibited significant association with breast cancer risk in Pashtun population of Khyber Pakhtunkhwa, Pakistan

**DOI:** 10.1002/hsr2.1846

**Published:** 2024-02-04

**Authors:** Wafa Sombal, Najeeb Ullah Khan, Bibi Maryam Khan, Muhammad Ismail, Mikhlid H. Almutairi, Samiullah Khan, Aakif Ullah Khan, Adeela Mustafa, Bushra Iftikhar, Ijaz Ali

**Affiliations:** ^1^ Institute of Biotechnology & Genetic Engineering (Health Division) The University of Agriculture Peshawar Peshawar Pakistan; ^2^ School of Life Science Jiangsu University Zhejiang Jiangsu Province People's Republic of China; ^3^ Department of Zoology Islamia College Peshawar Pakistan; ^4^ Zoology Department King Saud University Riyadh Saudi Arabia; ^5^ Institute of Radiotherapy and Nuclear Medicine (IRNUM) Peshawar Pakistan; ^6^ Department of Community Medicine Khyber Medical College Peshawar Pakistan; ^7^ Centre for Applied Mathematics and Bioinformatics (CAMB) Gulf University for Science and Technology Hawally Kuwait

**Keywords:** breast cancer, HER1&2, genetic polymorphism, genetic predisposition, risk association

## Abstract

**Background and Aims:**

Breast cancer is the most common type of cancer in women. The genetic polymorphism in HER (*HER1*–rs11543848 and *HER2*–rs1136201) were found to be associated with breast cancer risk in different ethnicities worldwide with inconsistent results. The aim of this research study was to evaluate the association of *HER1*–rs11543848 and *HER2*–rs1136201 polymorphisms as a risk of breast cancer in Pashtun population of Khyber Pakhtunkhwa, Pakistan.

**Methods:**

A total of 314 women including 164 breast cancer patients and 150 age and gender‐matched healthy controls were enrolled from June 2021 to May 2022. All the samples were subjected to DNA extraction followed by Tetra‐ARMS‐PCR for genotyping and gel electrophoresis.

**Results:**

Our results indicated that *HER1*–rs11543848 risk allele A (*p* = 0.0001) and heterozygous genotype GA (*p* = 0.0001) displayed highly significant association with breast cancer, while the homozygous mutant genotype AA indicated association but nonsignificant results (odds ratio [OR] = 2.637, 95% confidence interval [CI] = 1.2258–5.6756, *p* = 0.0833). Similarly, the *HER2*–rs1136201 risk allele G (*p* = 0.0023), the heterozygous genotype AG (*p* = 0.0530) and homozygous mutant genotype GG showed significant association (OR = 2.5946, 95% CI = 0.9876–6.8165, *p* = 0.0530) with breast cancer risk. Both the SNPs presented a higher but nonsignificant risk of breast cancer in postmenopausal women (OR = 2.242, *p* = 0.08 and OR = 2.009, *p* = 0.06). However, both the SNPs showed significant association (*p* < 0.005) with family history, metastasis, stage, luminal B, and TNBC.

**Conclusion:**

In conclusion, *HER1*–rs11543848 and *HER2*–rs1136201 polymorphisms are significantly associated with the higher risk of breast cancer in Pashtun population of Khyber Pakhtunkhwa, Pakistan. These findings advocate for further exploration with larger datasets, offering promising avenues for personalized approaches in breast cancer research and potentially enhancing clinical practices for better risk assessment and targeted management strategies.

## 1 INTRODUCTION

Breast cancer is the most common type of cancer and the leading cause of death in women worldwide.^1^ Among all the Asian countries, Pakistan has the highest incidence rate of breast cancer, usually one in every nine women has a lifetime risk of being diagnosed with breast cancer.^2^ There are several causes of breast cancer including lifestyle (diet, obesity, high alcohol, and lack of physical activity), ecological factors (exposure to radiations, and so on), and hereditary factors (genetics and family history) are the most significant risk factors.^3^

The growth factor receptors are involved in proliferation, differentiation, and survival of many tumor cells.^4^ A family of epidermal growth factor receptor (EGFR) containing tyrosine kinase domain plays an important role in cell growth, differentiation, and tumorigenesis.^5^ It consist of four structurally similar tyrosine kinase receptors; HER1 (erbB1 or EGFR), HER2 (erbB2), HER3 (erbB3), and HER4 (erbB4).^6^ The activation of these receptors regulates downstream pathways that help in cellular proliferation, cell differentiation, growth, and survival.^7^

*HER1* also known as *ErbB‐1* or *EGFR* is a transmembrane protein that causes the activation of downstream pathways resulting in cell division, growth, inhibition of apoptosis, and increasing angiogenesis after stimulation of the HER1 receptor.^8^ The extracellular domain of *HER1* gene consists of 1–14 Exons, the transmembrane region has Exon 15, while the intracellular domain possess 16–20 Exons.^9^ A ligand known as transforming growth factor alpha (TGF alpha) binds to the extracellular domain of *HER1* causing the auto phosphorylation of tyrosine kinase residue which is present in the intracellular region, due to this a downstream cascade is initiated.^7^ In *HER1* single nucleotide variation occurs at 497 codons in which the substitution of Arg (R) to Lys (K) occurs in the extracellular domain and is called R497K polymorphism (rs11543848). However, this mutation is detected in breast cancer as well as gliomas, colorectal and lung cancer.^10^, ^11^

*HER2* is a proto‐oncogene also known as *neu*‐oncogene which consists of 1255 amino acids and transmembrane glycoprotein (185 KD) that is located on the long arm of the chromosome 17 (17q12). *HER2 or ErbB2* is a member of EGFR that regulates the growth and differentiation of cells through several downstream pathways.^12^ The *HER2* receptor always exists in the form of monomer but when a ligand (HER3, HER4, and HER) binds to its extracellular domain it becomes dimerized causing the transphosphorylation of its intracellular tyrosine kinase domain. Various downstream signaling pathways which are mitogen‐activated protein kinase (MAPK), phosphatidylinositol‐4,5‐bisphosphate3‐kinase (P13K), and protein kinase C (PKC) are activated by *HER2* that helps in the regulation of cell growth and proliferation.^13^, ^14^

*HER2‐positive* breast cancer is highly aggressive and almost 20%–30% of breast cancer is due to the polymorphisms in *HER2* gene.^15^ The overexpression of *HER2* initiates an oncogenic signaling cascade by the activation of cytoplasmic tyrosine kinase activity.^16^ The polymorphisms in *HER2* enhance the tyrosine kinase activity as well as increasing the formation of *HER2* heterodimers that cause the progression of tumor.^17^ The polymorphism (I655V) present in the transmembrane region of *HER2* is linked with an increased risk of breast cancer.^18^ At codon 655, the conversion of Ile:ATC to V:GTC causes an increase in dimerization, tyrosine kinase activity, and auto‐phosphorylation leading to cell transformation.^19^ In *HER2‐positive* Breast cancer nearly 50 copies of *HER2* gene causes 40–100‐folds rise in HER2 proteins and approximately 2 millions of receptors of *HER2* are expressed on the surface of tumor cells.^20^ In contrast, trastuzumab is a monoclonal antibody that is used for the targeted therapy of HER2‐positive breast cancer improving overall survival.^21^ The correlation of Ile655Val (rs1136201) mutation in *HER2* with the higher risk of breast cancer was reported initially by Xie and his colleagues, However, it still remains controversial.^22^ The Ile655Val (rs1136201) polymorphism of *HER2* gene has been associated with breast cancer by many authors,^23^ while some other studies did not show its correlation with the development of breast cancer risk in women.^24^ Hence the overall results are inconsistent and controversial.

Due to controversies on *HER1* and *HER2* polymorphism association with breast cancer risk in various ethnicities, we aimed to conduct this study. We hypothesized that there could be an association of *HER1*–rs11543848 and *HER2*– rs1136201 with the risk of breast cancer in Khyber Pakhtunkhwa population. Therefore, the main aim of this study was to confirm the presence and association of *HER1* and *HER2* polymorphism with the risk of breast cancer.

## 2 MATERIALS AND METHODS

### 2.1 Patients’ enrollment and ethical approval

This case–control study was conducted during 2021–2022, containing 164 breast cancer patients and 150 age and gender‐matched healthy controls. All the participants were enrolled from the Institute of Radiology and Nuclear Medicine (IRNUM) Hospital Peshawar, Pakistan, who had only breast cancer with no other complications. Ethical approval was taken from the Institute of Biotechnology and Genetic Engineering (IBGE), The University of Agriculture Peshawar, Pakistan, and IRNUM hospital (IBGE, UAP/2022/002). A written informed consent was taken from all the participants after explaining the aim of the study. About 3 mL venous blood samples were taken in ethylenediaminetetraacetic acid tubes along with clinicopathological data. The blood was stored at −20°C for DNA extraction.

### 2.2 DNA extraction, SNP genotyping, and gel electrophoresis

The salting‐out extraction method was used for the isolation of genomic DNA already adopted in our lab.^25^ Briefly, red blood cells lysis was performed by lysis buffer (TKM1) and Triton‐X followed by incubation and centrifugation. The white blood cells were obtained in the form of pallet and nuclei lysis was done by TKM2 and Sodium dodecyl sulfate (SDS). After the incubation 6 M NaCl was added for the precipitation of protein. The Ethanol was used for the removal of excess salt and isopropanol was used for the precipitation of DNA. The extracted DNA was air‐dried and TE buffer was added for storage at −20°C until further use. The purity and concentration of DNA were determined by Nano drop (Thermo Fisher Scientific).

Amplification Refractory Mutation System PCR (ARMS‐PCR) was used for SNPs genotyping, used at our lab.^25^, ^26^ The specific outer and inner primer sequences (forward and reverse) were designed using online Primer blast software, to confirm homozygous and heterozygous alleles (Table 1). PCR mixture of 10 µL was prepared, including 5 µL master mix (5X) (Dream Taq, Thermo Fisher Scientific), 0.5 µL of each forward and reverse primers (1 µM of each primer), 3 µL ddH_2_O, and 1 µL of template DNA (50–100 ng). The amplified PCR products were confirmed with Gel electrophoresis using 1 KB DNA ladder on 2% agarose gel.

**Table 1:** Specific primers sequences designed for the detection of SNP genotype.

Genes	Primers	Sequences
*HER1* (rs11543848)	Forward outer	TTGTCCTTCCAGTCACGGTCG
	Reverse outer	TCAAACAGAATGCCTGTAAAGCT
	Forward inner	GGGGCCCGGAGCCCAA
	Reverse inner	GGCAAGAGACGCAGTCCC
*HER2* (rs1136201)	Forward outer	GAGCCAAGGCAGGTTTTAGAG
	Reverse outer	TCTGCGCCTGGTTGGG
	Forward inner	GCCCTCTGACGTCCATCG
	Reverse inner	GCCAACCACCGCAGAGAT

### 2.3 Statistical analysis

The collected data were statistically analyzed using software such as Statistical Package for Social Sciences (SPSS) version 16 to find the association of *HER1* and *HER2* genes polymorphisms with breast cancer risk. The clinicopathological, demographic characteristics, and genetic variants of breast cancer cases were detected by *χ* ^2^ test. The *p* < 0.05 was considered significant. Medcalc odd ratios calculator and 95% confidence intervals were used to figure out the possible association of *HER1* and *HER2* polymorphisms.

## 3 RESULTS

### 3.1 *HER1*–rs11543848 and *HER2*–rs1136201 polymorphism exhibited significant association with breast cancer patients

**Figure 1:**
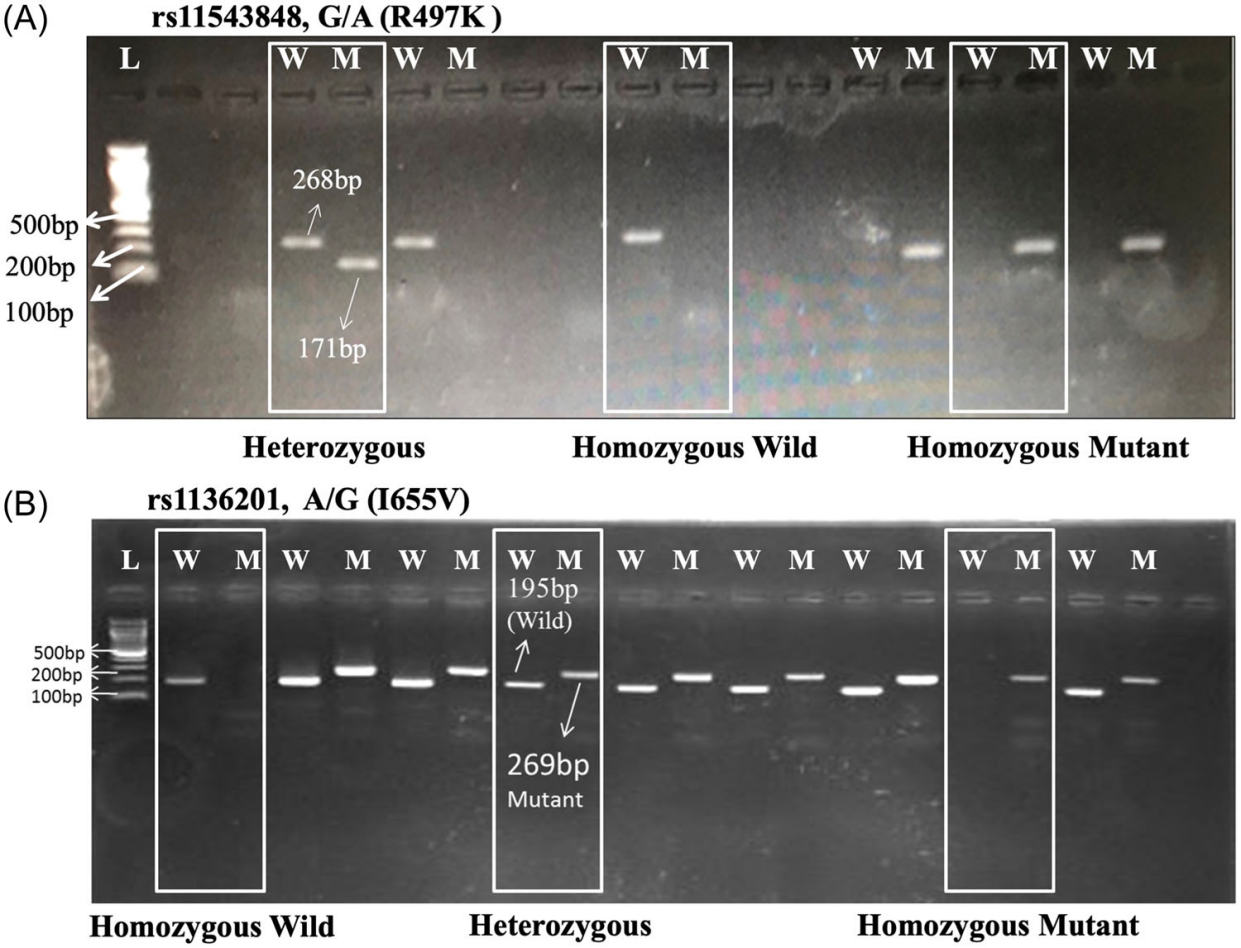
Representative electrogram of *HER1*–rs11543848 and *HER2*–rs1136201: (A) represents random samples for HER1–rs11543848, and (B) represents HER2–rs1136201. w, wild, m, mutant, and L, 100 bp DNA ladder (Thermo Fisher Scientific).

A total of 314 women including 164 breast cancer patients and 150 age and gender healthy controls of Pashtun population were enrolled in this study. All the samples were analyzed for SNP genotyping using T‐ARMS‐PCR protocol and confirmed with 2% agarose gel (Figure 1). Our results indicated that both the SNPs (*HER1*–rs11543848 and *HER2*–rs1136201) exhibited a significant association (*p* < 0.05) with breast cancer risk (Table 2). *HER1*–rs11543848 risk allele A (odds ratio [OR] = 2.7705, 95% confidence interval [CI] = 1.9830–3.8706, *p* = 0.0001) and heterozygous genotype GA (OR = 3.42, 95% CI = 2.1459–5.4506, *p* = 0.0001) displayed highly significant association with breast cancer, while the homozygous genotype AA showed association but nonsignificant results (OR = 2.637, 95% CI = 1.2258–5.6756, *p* = 0.0833). Similarly, *HER2*– rs1136201 risk allele G (OR = 1.8691, 95% CI = 1.3417–2.6038, *p* = 0.002) and the heterozygous genotype AG showed significant association (OR = 2.037, 95% CI = 1.2903–3.2159, *p* = 0.0023) with breast cancer but the homozygous genotype GG did not display significant association (OR = 2.5946, 95% CI = 0.9876–6.8165, *p* = 0.0530).

**Table 2:** Allelic and genotypic frequencies of *HER1*–rs11543848 and *HER2*–rs1136201polymorphism in breast cancer patients and healthy control.

Genotype/Allele	Control *n* (%) 150	Patients *n* (%) 164	Odd ratio	95% CI	*p V*alue
*HER1* (rs11543848)					
GG	79 (52.7)	24 (14.6)			
GA	61 (40.7)	144 (69.5)	3.42	2.1459–5.4506	*p* = 0.0001
AA	10 (6.7)	26 (15.5)	2.637	1.2258–5.6756	*p* = 0.0833
G	88 (73.3)	162 (49.3)			
A	32 (26.6)	166 (50.6)	2.7705	1.9830–3.8706	*p* = 0.0001
*HER2* (rs1136201)					
AA	69 (46.0)	38 (23.2)			
AG	75 (50.00)	110 (67.1)	2.037	1.2903–3.2159	*p* = *0.0023*
GG	6 (4.0)	16 (9.8)	2.5946	0.9876–6.8165	*p* = *0.0530*
A	86 (71.7)	186 (56.7)			
G	34 (28.3)	142 (43.3)	1.8691	1.3417–2.6038	*p* = 0.0002

### 3.2 Association of clinicopathological characteristics of breast cancer and *HER1*–rs11543848 and *HER2*–rs1136201 polymorphism

Clinicopathological characteristics of breast cancer such as age, family history, nulliparity, menstrual status, nodal status, metastasis, HER2 subtype, luminal A, luminal B, TNBC in patients were compared with *HER1*–rs11543848 and *HER2*–rs1136201 polymorphism. Based on the odd ratio and *p* value, our results revealed that *HER1*–rs11543848 displayed higher but nonsignificant association with the age group >45 years (OR = 1.6625, *p* = 0.2556), positive family history (OR = 7.400, *p* = 0.159), metastasis (*p* = 0.636), and luminal B positive patients (OR = 8.336). *HER2*–rs1136201 was significantly associated with family history (*p* = 0.0001), metastasis (*p* = 0.001), luminal B positive patients (OR = 14.82 *p* = 0.05), patients with age group >45 years (OR = 2.285, *p* = 0.030), and TNBC patients (OR = 3.5556 *p* = 0.001 (Table 3).

**Table 3:** Association of clinicopathological characteristics with *HER1*–rs11543848 and *HER2*–rs1136201 polymorphism.

Parameters	N (%)	*HER1*		OR	*p* Value	*HER2*		OR	*p V*alue
		GG	AA + GA			AA	GG + AG		
Age									
<45 years	78 (47)	14 (17.9)	64 (39.1)	0.601	0.255	24 (30.7)	54 (32.9)	0.437	0.030
>45 years	86 (52.4)	10 (11.6)	76 (46.3)	1.662		62 (70.0)	72 (43.9)	2.285	
Family history									
Negative	146 (89)	24 (16.4)	122 (74.3)	0.135	0.167	24 (16.4)	122 (74.3)	0.177	0.0001
Positive	86 (52.4)	0 (0.00)	18 (10.9)	7.400		14 (77.8)	4 (2.43)	0.056	
Nulliparity									
Negative	28 (17.0)	04 (14.2)	24 (14.6)	1.034	0.954	08 (28.5)	20 (12.1)	0.707	0.458
Positive	136 (82.9)	20 (14.7)	116 (70.7)	0.966		30 (22.1)	106 (64.6)	1.713	
Menstrual status									
Premenopausal	82 (50)	16 (19.5)	66 (40.2)	0.445	0.08	24 (29.3)	20 (12.1)	0.497	0.06
Postmenopausal	82 (50)	8 (9.8)	74 (45.1)	2.242		14 (17.1)	106 (64.6)	2.009	
Nodal status									
Negative	56 (34.1)	6 (10.7)	50 (30.4)	1.875	0.212	10 (17.8)	46 (28.0)	1.610	0.248
Positive	108(65.9)	18 (16.6)	90 (54.8)	2.533		28 (25.9)	80 (48.7)	0.621	
Metastasis									
Negative	116 (70.7)	16 (13.7)	100 (60.9)	1.250	0.636	36 (31)	80 (48.7)	0.096	0.001
Positive	48 (29.2)	8 (54.1)	40 (24.3)	0.800		2 (1.35)	46 (28.0)	10.35	
HER2 subtype									
Negative	112 (68.2)	16 (14.2)	96 (58.5)	1.090	0.853	30 (26.7)	82 (50.0)	0.417	0.117
Positive	52 (31.7)	8 (15.3)	44 (26.8)	0.916		8 (15.3)	44 (26.8)	2.012	
Luminal A									
Negative	54 (32.9)	10 (18.5)	44 (26.8)	0.641	0.326	8 (14.5)	46 (28.0)	0.912	0.08
Positive	110 (31.7)	14 (12.7)	96 (58.5)	1.558		14 (16.2)	72 (43.9)	0.916	
Luminal B									
Negative	144 (87.8)	24 (16.6)	120 (73.1)	0.115	0.13	38 (26.3)	106 (64.6)	0.667	0.05
Positive	20 (12.2)	0 (0.00)	20 (12.1)	8.336		0 (0.00)	20 (12.1)	14.82	
TNBC									
Negative	144 (69.5)	14 (12.2)	100 (60.9)	1.785	0.201	18 (15.7)	96 (58.5)	3.555	0.001
Positive	50 (30.4)	10 (20)	40 (24.3)	0.560		20 (40)	30 (18.25)	0.081	

Abbreviation: OR, odds ratio.

## 4 DISCUSSION

Breast cancer is the leading cancer in women worldwide. Many risk factors have been associated with the risk of breast cancer but recently due to the advent in molecular techniques, genetic predisposition of breast cancer have been extensively studied. Large number of studies have been conducted on *HER1* and *HER2* genes polymorphism and the risk of breast cancer on different populations. However, this research has revealed a landscape of conflicting and inconclusive findings, adding complexity to our understanding of these genetic factors’ direct impact on breast cancer susceptibility.^10^, ^24^, ^27^

Here in this study, we studied the association of *HER1*–rs11543848 and *HER2*–rs1136201 polymorphism with the risk of breast cancer in our population. A total of 314 women, containing 164 breast cancer patients and 150 age and gender‐matched healthy controls were analyzed for SNPs genotyping using Tetra‐ARMS‐PCR. Interestingly, our results indicated that both *HER1*–rs11543848 and *HER2*–rs1136201 risk alleles and their heterozygous genotypes are highly significant (*p* < 0.05) with breast cancer risk (Table 2).

Specifically, the risk allele A of HER1–rs11543848 (OR = 2.7705, 95% CI = 1.9830–3.8706, *p* = 0.0001) and the heterozygous genotype GA (OR = 3.42, 95% CI = 2.1459–5.4506, *p* = 0.0001) exhibited a notably significant association with breast cancer. However, the homozygous genotype AA showed an association without statistical significance (OR = 2.637, 95% CI = 1.2258–5.6756, *p* = 0.0833). Similarly, the risk allele G of HER2–rs1136201 (OR = 1.8691, 95% CI = 1.3417–2.6038, *p* = 0.002) and the heterozygous genotype AG displayed significant associations (OR = 2.037, 95% CI = 1.2903–3.2159, *p* = 0.0023) with breast cancer, whereas the homozygous genotype GG did not exhibit a significant association (OR = 2.5946, 95% CI = 0.9876–6.8165, *p* = 0.0530).

We further investigated the association between clinicopathological characteristics of breast cancer patients and these polymorphisms. Our analysis revealed that HER1–rs11543848 showed heightened but statistically nonsignificant associations with characteristics such as age group >45 years (OR = 1.6625, *p* = 0.2556), positive family history (OR = 7.400, *p* = 0.159), metastasis (*p* = 0.636), and luminal B subtype (OR = 8.336). In contrast, HER2–rs1136201 displayed significant associations with family history (*p* = 0.0001), metastasis (*p* = 0.001), luminal B subtype (OR = 14.82 *p* = 0.05), age group >45 years (OR = 2.285, *p* = 0.030), and TNBC patients (OR = 3.5556 *p* = 0.001), as outlined in Table 3.

Previously, a study was conducted in the Egyptian population which revealed that *HER1*–rs11543848 and *HER2*–rs1136201 are significantly associated with the increased risk of breast cancer, similar to our results. However, their results diverged in terms of clinicopathological parameters, where no association was found. This disparity might stem from differences in population demographics, genetic variations, or environmental factors, which could influence how these genetic variations manifest in breast cancer development.^10^ Contradictory findings were observed in comparison to a study on Tunisian patients^28^, which reported a nonsignificant association between HER1–rs11543848 polymorphism and breast cancer. Conversely, they identified an association with tumor grade and nodal status. Such discrepancies could arise due to variations in sample sizes, ethnic differences, or specific genetic makeup within these distinct populations, contributing to contrasting observations regarding the polymorphism's role in breast cancer development and progression.^28^ Similarly, another case–control study highlighted an association between AG + GG genotypes of HER2–rs1136201 and higher breast cancer risk in patients younger than 45 years old. However, our study presented an elevated association between genotypes of HER2–rs1136201 and patients over 45 years old. This disparity in age‐specific associations could be influenced by diverse genetic backgrounds or lifestyle factors prevalent in these age groups, emphasizing the complexity of genetic factors’ impact on breast cancer susceptibility across different age brackets.

Regarding the potential explanation for the association of HER2–rs1136201 polymorphism with breast cancer in our study, a hypothesis posits functional modifications rather than mere overexpression of HER2 protein. This suggests an increase in heterodimer formation, which could alter tyrosine kinase activity. This theory underscores the need for further mechanistic studies to elucidate how these genetic variations lead to functional changes, impacting breast cancer susceptibility and disease progression.

It is important to note the inherent variability in somatic changes related to these polymorphisms across diverse ethnic groups, influencing their roles in breast cancer. This divergence underscores the complexity of genetic factors and necessitates cautious interpretation when comparing studies conducted in distinct ethnic populations, as genetic variations and their associations with breast cancer can vary significantly.

In essence, the differing outcomes in these studies emphasize the intricate interplay between genetic variations, environmental factors, and ethnic diversity in shaping the role of HER1–rs11543848 and HER2–rs1136201 polymorphisms in breast cancer susceptibility and clinicopathological characteristics. These complexities warrant further comprehensive research to unravel the precise mechanisms underlying these associations and their implications across diverse populations.

## 5 CONCLUSION

In conclusion, both *HER1*–rs11543848 and *HER2*–rs1136201 polymorphism exhibited significant association with breast cancer risk in the Pashtun population of Khyber Pakhtunkhwa, Pakistan. These findings hold promise for advancing personalized medicine by potentially enabling more targeted screening and intervention strategies for individuals within this specific demographic. Moreover, they underscore the need for continued research in exploring these genetic variations’ broader implications across different populations, paving the way for tailored preventive measures and more effective therapeutic interventions in the realm of breast cancer management. Such insights could ultimately contribute to improved patient outcomes and more precise clinical approaches in combating this prevalent disease.

## AUTHOR CONTRIBUTIONS

**Wafa Sombal**: Investigation; writing—original draft; validation; data curation. **Najeeb Ullah Khan**: Conceptualization; funding acquisition; methodology; validation; visualization; writing—review & editing; formal analysis; project administration; supervision; resources; data curation; software; investigation; writing—original draft. **Bibi Maryam Khan**: Formal analysis; data curation. **Muhammad Ismail**: Methodology; visualization; formal analysis; data curation. **Mikhlid H. Almutairi**: Funding acquisition; writing—review & editing; formal analysis; software; data curation. **Samiullah Khan**: Conceptualization; writing—review & editing; data curation. **Aakif Ullah Khan**: Conceptualization; writing—review & editing; formal analysis. **Adeela Mustafa**: Funding acquisition; formal analysis; visualization. **Bushra Iftikhar**: Funding acquisition; visualization; formal analysis. **Ijaz Ali**: Conceptualization; writing—review & editing; formal analysis.

## CONFLICT OF INTEREST STATEMENT

The authors declare no conflict of interest.

## ETHICS STATEMENT

Ethical approval was taken from the Institute of Biotechnology and Genetic Engineering (IBGE), The University of Agriculture Peshawar, Pakistan under (IBGE, UAP/2022/002). A written informed consent was taken from all the participants after explaining the aim of the study.

## TRANSPARENCY STATEMENT

The lead author Najeeb Ullah Khan affirms that this manuscript is an honest, accurate, and transparent account of the study being reported; that no important aspects of the study have been omitted; and that any discrepancies from the study as planned (and, if relevant, registered) have been explained.

## Data Availability

All the necessary data are included in the manuscript, related data will be provided on request from corresponding author.
